# Biomarkers of Acute Kidney Injury after Cardiac Surgery: A Narrative Review

**DOI:** 10.1155/2019/7298635

**Published:** 2019-06-27

**Authors:** Binbin Wu, Jianghua Chen, Yi Yang

**Affiliations:** ^1^Kidney Disease Center, The First Affiliated Hospital, College of Medicine, Zhejiang University, Hangzhou, China; ^2^Key Laboratory of Kidney Disease Prevention and Control Technology, Zhejiang Province, China; ^3^National Key Clinical Department of Kidney Diseases, Hangzhou, China; ^4^Institute of Nephrology, Zhejiang University, Hangzhou, China; ^5^The Third Grade Laboratory under the National State, Administration of Traditional Chinese Medicine, Hangzhou 310003, China

## Abstract

Cardiac surgery-associated acute kidney injury (CSA-AKI) is a major and serious complication in patients undergoing cardiac surgery and is independently associated with perioperative mortality and mortality. Therapeutic intervention aiming at reversing kidney dysfunction seems disappointing across multiple settings. Consequently, attention has shifted from treatment to prevention and early detection. The Kidney Disease: Improving Global Outcomes (KDIGO) guidelines have unified diagnostic standards mainly based on the serum creatinine (Scr) level or urine output, but neither marker is kidney specific. Efforts have been made to identify novel biomarkers with high sensitivity and specificity. The diagnostic capabilities of neutrophil gelatinase-associated lipocalin (NGAL) and G1 cell cycle arrest biomarker as biomarkers have been confirmed in a large number of clinical trials. The utility of biomarkers of cardiac function and inflammation has been validated in clinical studies. Aiming to offer valuable information for further research, we summarize the progress in defining current markers relevant to CSA-AKI in the last three years.

## 1. Introduction

Cardiac surgery-associated acute kidney injury (CSA-AKI) is a common and serious complication of cardiac surgery [1]. More than 2 million people worldwide undergo cardiac surgery each year [2] and the incidence of CSA-AKI varies from 5% to 42% in different settings and regions [3–5]. CSA-AKI is independently associated with serious adverse outcomes, increased perioperative mortality, prolonged hospital stay, and increased cost of care [2, 6, 7]. Many therapeutic interventions have been performed to reverse established AKI; however, the effects have been disappointing. With no effective treatment being developed, attention has shifted from treatment to prevention and early detection [8–10].

It seems that no consensus has been reached on the definition of CSA-AKI. Over 35 different definitions have been described in the literature [11] since the concept of acute kidney failure was defined by Dr. Sminth in 1946 [12]. Based on tremendous research, the Acute Dialysis Quality Initiative first classified acute kidney injury (AKI) into five stages in 2004: risk, injury, failure, loss, and end-stage renal disease (RIFLE) [13]. Five years later, the Acute Kidney Injury Network published a statement defining AKI on the basis of the serum creatinine (Scr) level and urine output [14]. In 2012, the Kidney Disease: Improving Global Outcomes (KDIGO) group [15] proposed a guideline that successfully standardized AKI screening and divided AKI into 3 stages (Table 1). Patients who had cardiac surgery and fulfill the KDIGO criteria within 7 days postoperation can be said to have CSA-AKI [16].

Compared with RIFLE criteria, the KDIGO criteria could detect more patients with AKI in critically ill patients [17, 18]. However, there still have been some pitfalls. The KDIGO definition of AKI is mainly based on changes in the urine output (<0.5 ml/kg/h for at least 6 hours) or Scr level (increased by ≥26.5 *μ*mol/l at least), which are not kidney specific [10]. Oliguria is a response to intravascular hypovolemia. It exists not only in acute tubular necrosis but also in the urinary tract and can be shock induced by various etiologies [19]. The concentrations of Scr may take 24-36h to rise after a definite renal insult [20]. In addition, creatinine levels are often influenced by factors from the prerenal azotemia and body (sex, weight, muscle metabolism, body volume, food, and drugs) [21].

**Figure 1:**
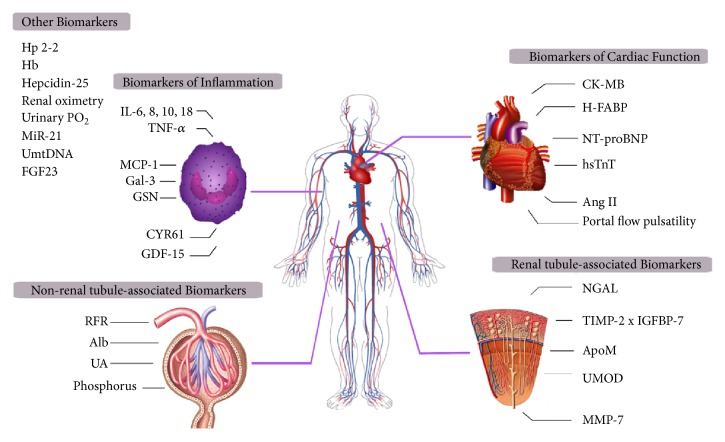
Biomarkers of cardiac surgery-associated acute kidney injury (CSA-AKI). Biomarkers of CSA-AKI are divided into four categories including renal tubule-associated biomarkers, nonrenal tubule-associated biomarkers, biomarkers of cardiac function, and biomarkers of inflammation. Other biomarkers that cannot be classified into the above types are listed as one category at the top left.* CK-MB:* creatine kinase-MB,* H-FABP:* heart fatty acid binding protein,* NT-proBNP:* N-tnd persistederminal prohormone of brain natriuretic,* AngII:* angiotensin II,* NGAL:* neutrophil gelatinase-associated lipocalin,* TIMP-2:* tissue inhibitor of metalloproteinases 2,* IGFBP-7:* insulin-like growth factor-binding protein 7,* ApoM:* apolipoprotein M,* UMOD:* urinary uromodulin,* MMP-7:* matrix metalloproteinase-7,* IL:* interleukin,* TNF-α:* tumor necrosis factor alpha,* MCP-1:* monocyte chemotactic protein-1,* Gal-3*: galectin-3,* GSN:* gelsolin,* CYR61:* cysteine-rich protein 61,* GDF-15:* growth differentiation factor 15,* RFR:* renal functional reserve,* Alb:* albumin,* UA:* uric acid,* Hp2-2:* 2-2 phenotype of haptoglobin,* Hb:* hemoglobin,* PO*_*2*_*:* oxygen tension,* MiR-21:* microRNA-21,* UmtDNA:* urinary mitochondrial DNA, and* FGF23:* fibroblast growth factor 23.

Given the limitations of Scr and urinary output, efforts have been made to identify biomarkers that could serve as “renal troponin.” Ideally, such biomarkers would identify possible mechanisms of renal injury and provide risk stratification and prognostic information [21]. In this review, we summarize the progress in defining novel biomarkers relevant to CSA-AKI in the last three years (Figure 1) and categorize them into five types according to their clinical utility (Table 2), aiming to offer valuable thinking for further research.

## 2. Renal Tubule-Associated Biomarkers 

### 2.1. NGAL (Neutrophil Gelatinase-Associated Lipocalin)

NGAL, which belongs to the superfamily of lipocalins, is a small 25-kDa protein with 178 amino acids [22]. Produced by neutrophils, plasma NGAL is freely filtered through the glomeruli and then totally reabsorbed by the proximal tubules [23]. NGAL reduces apoptosis and increases proliferation in kidney tubule cells through processes involving immune modulation, inflammation, and neoplastic transformation [24]; thus, NGAL plays a critical physiological role in renal ischemia. A consensus has also been reached that urinary NGAL after kidney injury is mainly expressed after kidney injury by the thick ascending limb and collecting ducts of the nephron but also by epithelial cells.

In preclinical and initial clinical testing stages, NGAL demonstrated excellent performance in the early detection of postoperative AKI and was considered the “troponin” of the kidneys [25–27]. Mishra J et al. [28] first reported increases in the NGAL level in the serum and urine among 71 children who underwent cardiac surgery. A nearly 10-fold increase in the urine and plasma levels within 2 h was identified in the patients who subsequently developed AKI. Since then, data regarding NGAL measurements have been accumulating [29]. Plasma and urine NGAL have emerged as highly predictive early biomarkers of AKI with areas under the receiver-operating characteristic curves (AUROCs) of 0.91 and 0.998, respectively. The great diagnostic power of these levels was also confirmed in other types of population (adults and critically ill patients) and other clinical situations (intensive care units (ICU) and cardiopulmonary bypass surgery), with AUCs ranging from 0.71 to 0.85 [30–32]. In addition, increased levels could be detected in the early postoperative period, appearing as soon as ICU admission [33–35]. Predictive accuracy does not seem to be affected by preoperative renal function. It was documented that the diagnosis of AKI could be made 20 h earlier using plasma NGAL at 6 h rather than Scr in adults with chronic kidney failure (CKD) [35], even in patients who underwent coronary artery bypass grafting (CABG) surgery [36]. Though confirmed to have high sensitivity and speciality for its diagnostic capability, the application of NGAL in predicting clinical outcome is limited. Mercede et al. [37] hypothesized that urinary NGAL levels may represent only a renal response to cardiac surgery. Jowita et al. [38] reported that urinary NGAL could not predict 30-day or 5-year mortality.

In the past few years, interest has been aroused in the role of biomarker combinations in the early detection of AKI after cardiac surgery [37, 39]. Oguzhan et al. [40] noted that the measurement of urinary NGAL and serum cystatin C (CysC) levels may detect AKI earlier than blood urea nitrogen and Scr in diabetic patients. A retrospective study of 345 children enrolled from 2004 to 2007 showed that composites of urinary NGAL and plasma CysC, measured at 2 h after CPB initiation, are superior to Scr for predicting severe AKI (likelihood 34.2 versus 3.8, respectively) [41]. A prospective observational study was carried out to assess the efficacy of combinations of urinary NGAL and kidney injury molecule-1 (KIM-1). The results demonstrated that urinary NGAL was relatively sensitive while KIM-1 seemed to be specific to ischemic renal injury (IRI), and a combination of the two markers yielded an AUC of 0.906 [42]. It has been reported that serum chitinase 3-like protein 1 combined with NGAL has a good predictive value for severe AKI (AKI stage≥2) [43]. Additionally, combining urinary NGAL with urinary CysC, KIM-1, chemokine ligand 2, and interleukin- (IL-) 18 enables the early identification of patients with serious adverse outcomes (hospital mortality or renal replacement therapy (RRT)) [44].

Recently, researchers described an issue with biomarker combinations. Gunnar et al. [45] reported that the combination of multiple biomarkers (NGAL, CysC, and liver fatty acid-binding protein) does not significantly improve diagnostic power in adults following CPB. John et al. [46] found that the combination of urinary NGAL and other biomarkers fails to exhibit discriminant utility in high-risk patients compared to the best single biomarker. Meisner et al. [47] evaluated the performance of biomarker combinations in the Translational Research Involving Biomarkers and Endpoints (TRIBE) cardiac surgery cohort. Researchers found that all published reports are susceptible to failing to acknowledge potential bias, especially in the ICU. Resubstitution bias, model selection bias, and bias owing to center differences may prevent the development of biomarker combinations [47]. To further study the associations between individual or combined biomarkers and AKI, Hilde et al. [48] conducted a systematic review of adult cardiac surgery patients. It is worth mentioning that the authors recommended the incorporation of new biomarkers for AKI to decrease the sample size and lower the cost of trials [49]. Therefore, rigorous study design, analysis, and reporting of biomarker combinations are supposed to be of great importance in future studies before the realization of the promise of biomarkers in clinical practice.

### 2.2. G1 Cell Cycle Arrest Biomarker

G1 cell cycle arrest is a protective mechanism, reducing oxygen consumption and preventing the division of cells with damaged DNA, thereby protecting the kidneys from extended exposure to stress and injury [50]. Tissue inhibitor of metalloproteinases 2 (TIMP-2) and insulin-like growth factor-binding protein 7 (IGFBP7), G1 cell cycle arrest proteins synthesized in and secreted by renal tubular epithelial cells, are found to accumulate in the urine after IRI. Previous clinical studies assessed the utility of [TIMP-2] x [IGFBP-7] as independent predictors of AKI [51]. Intending to translate an encouraging finding into a diagnostic tool for the risk assessment of AKI, the Food and Drug Administration approved NephroCheck in the U.S. in 2014. Since then, an increasing number of studies have been carried out in patients undergoing cardiac surgery.

There is no standardized cutoff for the measurement of [TIMP2] x [IGFBP7]. Fabian et al. [52] recruited 40 patients undergoing transcatheter aortic valve implantation (TAVI) enrolled in a prospective observational trial and evaluated the diagnostic accuracy of [TIMP-2] x [IGFBP-7] in the prediction of AKI. When applying the cutoff of 1.03, the AUC was determined to be 0.971, and the utility of [TIMP-2] x [IGFBP-7] was confirmed. Anna et al. [53] agreed that [TIMP-2] x [IGFBP-7] can be used to identify patients at increased risk of AKI, but the authors also illustrated that a cutoff of 2 was not confirmed in their cohort study. Despite the various reports, a value> 0.3 seems to be acceptable [54–56]. Chiara et al. [55] used the NephroCheck system to measure the urinary concentration of [TIMP-2] x [IGFBP-7] and proposed that physicians should perform therapeutic interventions when the result is >0.3 ng/dL. When applying the >0.3 cutoff to predict KDIGO stage 2 or 3 AKI, the AUC of 0.82 has 100% sensitivity and 100% negative predictive value [56].

No agreement about time frame was reached so far. Results of a multicentre, prospective cohort study performing in infants showed that [TIMP-2] x [IGFBP-7] correlated with subsequent AKI following CPB. Urinary samples were collected before surgery and 1, 4, and 24 h after CPB initiation. Researchers recommended measuring samples at 1 h as a supplement to traditional criteria [57]. Increased levels have been detected as early as 4 h after CABG or ICU admission, and these levels exhibit a better performance in the prediction of moderate to severe AKI [58, 59]. The results of a randomized controlled trial showed that patients with a 10-fold increased level at 3 h postoperation had a 650% increased risk of AKI progression [60]. Patients who developed postoperative AKI had a markedly higher concentration of [TIMP-2] x [IGFBP-7] at 12 h after CPB initiation, with a threefold higher odds ratio of AKI progression than those who did not suffer renal insufficiency [60].

Urinary [TIMP-2] x [IGFBP-7] which shows good sensitivity and specificity seems to be a promising predictor of AKI and clinical outcome [27, 61, 62]. A prospective study regarding pediatric cardiac surgery validated the utility of the urinary [TIMP-2] x [IGFBP-7] level measured at 4 h postoperation, showing an AUROC of 0.85 [63]. Miao et al. [64] performed a meta-analysis of cardiac surgery including four prospective cohort studies (a total of 277 patients). Based on available data from inception to December 25, 2016, urinary [TIMP2] x [IGFBP7] performs well in identifying patients who need RRT (AUROC 0.915). Its predictive ability for 28-day mortality was confirmed in a prospective study with an AUROC of 0.77 [65]. Moreover, [TIMP2] x [IGFBP7] does not seem to be interfered with by age or underlying chronic disease. Michael et al. [66] analyzed data and constructed AUROC for critically ill patients from two multicenter studies. They found no significant attenuation in the predictive ability of cell cycle arrest biomarkers despite the presence of CKD, diabetes mellitus, or congestive heart failure.

### 2.3. Novel Renal-Tubule Associated Biomarkers

Damage to the kidney proximal tubules is considered one of the pathophysiological mechanisms of the development of AKI [62]. Several small proteins reflect impaired proximal tubular function and have attracted attention in recent years. Uromodulin (UMOD) is a 95-kDa glycoprotein positively correlated with the estimated glomerular filtration rate and tubular function. Pranav et al. [67] conducted a post hoc analysis of a prospective study that enrolled adults undergoing on-pump cardiac surgery. They found that the patients with lower uromodulin-to-creatinine ratios were more likely to develop AKI and have higher peak Scr levels than the patients with higher ratios. Michael et al. [68] found that children with the lowest concentration of preoperative urinary UMOD had the highest risk of AKI, which implied that urinary UMOD levels could be used to stratify high-risk patients and minimize adverse outcomes. Urinary apolipoprotein M, demonstrating an AUC of 0.70 with a cutoff level of 1.45 nmol/L, is assumed to be a biomarker of AKI in children undergoing heart surgery [69]. Matrix metalloproteinase-7 (MMP-7), a member of a family of zinc-containing enzymes, localizes in the renal tubular epithelium. Faithfully reflecting the activity of renal Wnt/b-catenin induced in AKI, MMP-7 is easily excreted into the urine after renal insults [63, 70]. Yang et al. [71] performed a prospective multicenter cohort study of 323 children and 398 adults undergoing cardiac surgery test for the diagnostic ability of MMP-7. Urinary MMP-7 levels peaked within 6 h, and urinary MMP-7 had AUCs of 0.81 (children) and 0.76 (adults) in patients developing severe AKI, outperforming a clinical model and urinary biomarkers (NGAL, IL-18, and [TIMP-2] x [IGFBP-7]). The above molecule might be used as noninvasive biomarker of renal injury, and more work needs to be done to further confirm this potential.

## 3. Nonrenal Tubule-Associated Biomarkers

Reductions in the glomerular filtration rate lead to electrolyte imbalance, the sudden accumulation of uremic toxins, and subsequent acidemia and contribute to impaired renal function [72]. Attention has been given to the glomerular filtration dysfunction in AKI in adults [73]. It is recognized that glomerular filtration is closely related to potential kidney function and used for the assessment of renal recovery after AKI [74, 75]. Researchers have started to explore the relationship between glomerular dysfunction and AKI.

Increased levels of molecular markers (urinary albumin (Alb), uric acid (UA), and phosphorus) are thought to be associated with impaired kidney function. A prospective observational study including adolescents undergoing repairs for congenital heart disease with CPB indicated that uAlb is a useful marker of AKI, as determined by the RIFLE criteria [76]. The level of urinary Alb measured at ICU admission significantly differed between AKI patients and non-AKI patients (13.5 vs 6.0, respectively). Recent data suggested that serum UA might be an important factor in the pathogenesis of AKI in addition to its role in the setting of tumor lysis [77]. Ahsan et al. [78] conducted a systematic analysis of current evidence and concluded that the serum UA level at 1 h postoperation is capable of predicting a subsequent increase in the urinary NGAL or SCr level, which implies impaired renal function. A prospective observational study further explored the role of UA in patients undergoing open heart surgery. The serum UA level measured at 24 hours after surgery had a larger AUROC than the urinary NGAL level, demonstrating the utility of serum UA predicting the progression of AKI and the need for RRT [79]. In the last few months, Kaufeld et al. [80] validated a correlation between the preoperative serum UA level and postoperative AKI. Preoperative serum UA appears to be an independent risk indicator of AKI patients than in patients who do not suffer renal dysfunction. Researchers have investigated the relationship between serum phosphorus and AKI. The AUROC for the serum phosphorus level at 24 h was found to be 0.84 with a cutoff value of 6.4 mg/dL, demonstrating its predictive role as an early indicator of AKI in pediatric cardiac surgery patients [81]. In addition, renal functional reserve (RFR) has also attracted attention. Faeq et al. [74] found that adults with an RFR no higher than 15 mL· min^−1^· 1.73 mL^−2^ had a nearly twelve-fold increased risk of developing AKI after cardiac operation. If the above markers are confirmed in larger multicenter studies, physicians will be able to take preventive measures at an early stage. Clinical trials and prospective cohort studies are warranted to determine the significance of these markers in the future.

## 4. Biomarkers of Cardiac Function

Activation of the immune, sympathetic nervous systems (SNS) and renin-angiotensin-aldosterone systems has been referred to as “cardio-renal connectors” between the kidneys and heart [72, 82]. In the context of AKI in animal models, the secretion of pro- and anti-inflammatory cytokines combined with neutrophil infiltration mediates structural and functional changes in myocardial cells in response to renal ischemia and reperfusion injury [83]. SNS activation directly affects intrarenal hemodynamics and stimulates angiotensin (Ang) II release [84]. Increased levels of Ang II modify the myocardial structure, further promoting cardiac myocyte apoptosis and activating proinflammatory pathways [85, 86]. This organ-organ interaction implies that cardiac markers that reflect myocardial injury may serve as predictors of AKI.

Creatine kinase-MB (CK-MB), an indicator reflecting the severity of the underlying heart disease, has been found to be associated with an increased risk of postoperative AKI [87]. An analysis of the data from TRIBE-AKI demonstrated the predictive capability of preoperative CK-MB. Children developing AKI had higher preoperative median levels than those in non-AKI groups (P<0.1). With an AUROC of 0.77, CK-MB was confirmed to provide good discrimination and improve reclassification [88]. Belonging to a family of highly conserved proteins, heart fatty acid binding protein (H-FABP) is widely expressed in the cytosol of myocardial cells and was discovered as a myocardial protein that could indicate renal injury. The preoperative H-FABP level provides information on diagnosis with a relatively high AUC (0.7) in patients following cardiac surgery [88]. Jennifer et al. [89] reported that both the preoperative log(H-FABP) and first postoperative log(H-FABP) were associated with all stages of AKI. After adjusting for confounding factors, the associations still remained significant.

As the only marker independently associated with severe AKI, N-tnd persistederminal prohormone of brain natriuretic (NT-proBNP) predicts poor patient outcome. Patients with obvious increases in their NT-proBNP level within 6 h postoperation have a nearly twenty-fold increased risk of one-year mortality [90]. The risk-stratifying usefulness of NT-proBNP was examined in a multimarker study that evaluated the preoperative risk assessment of AKI. The addition of NT-proBNP to the clinical model provided an incremental increase in predictive utility (AUROCs ranging from 0.81 to 0.31) and powerfully improved the model predictions, with 12% of patients being reclassified correctly [91]. The high-sensitivity troponin T (hsTnT) level was found to rise immediately in the settings of cardiac surgery-induced AKI and was sustained over the course of the entire study [92]. Performing a retrospective cohort study, William et al. [93] reported that the patients with detectable portal flow pulsatility were much likely to suffer renal injury, suggesting that doppler ultrasound might be a useful tool to detect high-risk patients in the setting of cardiogenic venous congestion. A cohort study constructed with a control group suggested that the plasma levels of Ang II both decreased over time in AKI groups, but the decrease did not reach statistical significance [94].

## 5. Biomarkers of Inflammation

Putative conclusions were reached that inflammation plays a key role in the pathophysiology of AKI and that oxidative stress and hemolysis form pathways complementary to inflammation [95]. The CPB pump is considered to be the culprit in the activation of inflammation during cardiac surgery, resulting in an obvious elevation in the levels of proinflammatory cytokines [96]. Evidence from experiments has demonstrated that the kidneys are likely to suffer endothelial dysfunction caused by circulating cytokines and activated neutrophils [97].

As shown in clinical studies, increases in the postoperative levels of plasma proinflammatory cytokines, such as IL-18, IL-6, IL-10, and IL-8, are associated with a subsequent AKI. The most frequently analyzed inflammatory factor in the context of AKI is IL-18, an 18-kDa proinflammatory cytokine detected in the urine after ischemic tubular damage. A postanalysis of the data from the TRIBE cohort study assessed the predictive values of five urinary biomarkers in the early detection of AKI. The authors reported that an increased level of IL-18 at 0-6 h postoperation was an independent risk factor for greater odds of severe AKI (OR 1.22) [98]. Chirag et al. [49] reported that the combination of IL-18 and NGAL might improve the risk stratification of patients who experienced AKI progression. In a prospective pilot study enrolling patients undergoing TAVI, the changes in the urinary IL-18 level at 2, 4, and 12 h following surgery did not reach significant differences after adjusting for the Society of Thoracic Surgeons risk factors [99]. Jason et al. [97] performed a substudy of the TRIBE-AKI data including 106 children (excluding infants age <10 months) to assess the associations between the plasma IL-6 and IL-10 levels and AKI. The results demonstrated that the highest tertile preoperative IL-6 level was associated with increased odds of stage 2/3 AKI (OR 6.41) and a longer hospital stay. William et al. [100] validated the hypothesis that plasma IL-6 and IL-10 have potential as biomarkers for perioperative outcomes and published their results in the Journal of the American Society of Nephrology. When categorized into tertiles, elevated IL-6 expression demonstrated a strong association with the risk of AKI in a dose-dependent manner (with second tertile OR 1.61; third tertile adjusted OR, 2.13). Adult patients with elevated postoperative IL-10 levels had a twofold increased risk of developing AKI. In addition, Christina et al. [101] reported that the preoperative IL-8 level and postoperative tumor necrosis factor alpha (TNF-*α*) level could identify people at risk of suffering AKI before changes in the Scr level, demonstrating a potential to detect AKI early in the setting of pediatric cardiac surgery.

The plasma monocyte chemotactic protein-1 (MCP-1) level is markedly upregulated after cardiac operations. Patients with AKI tend to have a higher level of MCP-1 and a higher risk of death within 3 years of follow-up than non-AKI patients [102]. Moritz et al. [103] proposed adding circulating Galectin-3 to a clinical model to improve discriminatory power. A prospective study enrolling infants and young children after CPB reported that the joint use of CPB time and the plasma gelsolin (GSN) level at 6 h postoperation presented an excellent predictive value for AKI [104]. Osama et al. [105] discovered a marked increase in the serum level of cysteine-rich protein 61 (CYR61), an extracellular matrix molecule abundant at sites of inflammation, in patients developing more severe kidney injury. Charles et al. [106] reported that the preoperative level of growth differentiation factor 15 (GDF-15), a cytokine upregulated during inflammation or oxidative stress, not only improved the ability to predict mortality after CABG surgery but also performed better than the European system for cardiac operative risk evaluation in predicting AKI. A similar conclusion was reached in an observational cohort study (AUROC of 0.75) [11]. The above inflammatory cytokines would benefit early therapeutic interventions and decrease medical costs if their utilities in identifying high-risk patients are validated.

## 6. Other Biomarkers 

Fibroblast growth factor 23 (FGF23) is a bone-derived hormone that regulates phosphorus and vitamin D metabolism. Mark et al. [107] reported that the C-terminal FGF23 level increases by 2 h postreperfusion and remains elevated through 24 h postreperfusion in pediatric cardiac surgery patients, and its predictive ability was examined in another study and found to have an AUC of 0.74. Moreover, the FGF23 level is considered an independent predictor of postoperative complications. The results of a prospective study showed that a higher circulating postoperative FGF23 level contributes to an increased risk of the need for RRT or of hospital death. Oded et al. [108] suggested using FGF23 with clinical factors to enhance risk prediction.

The type and concentration of hemoglobin (Hb) seem to play roles in the early detection of AKI. Feng et al. [109] found that the 2-2 phenotype of haptoglobin displayed a good predictive value for AKI in patients with diabetes mellitus. Another prospective observational cohort study examined the utility of postoperative urine hepcidin-25 as a diagnostic tool and identified that its inclusion improved a clinical AKI prediction model compared with the clinical model alone [110]. The French national TAVI registry illustrated the potential utility of preoperative anemia in the early detection of AKI [111]. Additionally, postoperative anemia is thought to be an independent risk indicator. Researchers involved in a Spanish multicenter cohort study [112] reported that patients with a postoperative hemoglobin (Hb) level <19 g/dL tended to experience CSA-AKI (OR 1.41) after adjusting for intraoperative red blood cell transfusion. An Hb drop after cardiac surgery also contributed to a prolonged hospital length of stay [113]. In addition, iron metabolism may play a role in the pathophysiology of kidney injury. A prospective cohort identified that, compared with patients with a low level, patients with an elevated plasma catalytic iron level had greater odds of AKI and adverse postoperative outcomes [114].

The results of a case-control study that enrolled infants undergoing CABG demonstrated that intraoperative low renal oximetry values correlate with the occurrence of AKI and might be superior to NGAL and Scr [115]. Zhu et al. [116] proposed using a urinary catheter catheterized to monitor renal oxygenation after his finding that a low urinary PO_2_ (oxygen tension) was independently associated with postoperative AKI. Recent studies have identified plasma microRNA-21 (miR-21), which mediates glomerulosclerosis and podocyte apoptosis, as an important new biomarker. Luise et al. [117] reported that a low baseline miR-21 level (especially <0.31) predicted severe AKI with an AUROC of 0.701, suggesting that miR-21 could be used in preprocedural risk assessment. Arvin et al. [118] reported that urinary miR-21, especially when the level is measured at 6 h after surgery, is more reliable than serum miR-21. Animal models have been constructed to evaluate urinary mitochondrial DNA (UmtDNA) as a marker of renal injury [119]. If confirmed in clinical practice, it may provide mitochondria-targeted therapies for AKI.

## 7. Conclusions

Great progress, the result of emerging proteomic technology over the past decades, has been made in the early detection of CSA-AKI. The diagnostic utility of renal tubule-associated biomarkers, such as NGAL and G1 cell cycle arrest biomarkers, has been validated in innumerable clinical trials. Biomarkers of cardiac function and inflammation seem to be indicators that help characterize the pathophysiology of disease. Other biomarkers show potential for improving risk stratification. Though capable of characterizing the cause and course of renal insufficiency, the individual utilization of biomarkers needs to be confirmed by large multicenter clinical trials.

## Figures and Tables

**Table 1:** KDIGO criteria for the classification of AKI.

Stage	Serum creatinine level	Urine output
1	Increase of ≥0.3 mg/dl (≥26.5 *μ*mol/l) within 48 h or increase of 1.5–1.9-fold over baseline within 7 days	< 0.5 ml/kg/h for 6 to 12 h
2	Increase of 2.0–2.9-fold over baseline	< 0.5 ml/kg/h for 12 h
3	Increase of 3.0-fold over baseline, increase of ≥4.0 mg/dl (≥353.6 *μ*mol/l), initiation of renal replacement therapy, or a GFR decrease < 35 ml/min/1.73 m^2^ for patients < 18	< 0.3 ml/kg/h for 24 h or anuria for 12 h

KDIGO: Kidney Disease Improving Global Outcomes; AKI: acute kidney injury; GFR: glomerular filtration rate.

**Table 2:** Clinical characteristics of biomarkers relevant to CSA-AKI.

	AKI detection preoperative	AKI detection postoperative	Helpful for diagnosis	Severity prediction	Mortality prediction
Non-renal tubule-associated biomarkers	RFR UA	Alb phosphorus	RFR, Alb phosphorus	UA	

Renal tubule-associated biomarkers	UMOD	NGAL [TIMP-2]x [IGFBP-7] ApoM, MMP-7	NGAL [TIMP-2]x [IGFBP-7] UMOD	MMP-7 [TIMP-2]x [IGFBP-7] ApoM	MMP-7

Biomarkers of cardiac function	CK-MB H-FABP NT-proBNP	hsTnT portal flow pulsatility AngII	CK-MB, hsTnT H-FABPv NT-proBNP portal flow pulsatility	AngII	AngII

Biomarkers of inflammation	IL-6, 8 MCP-1 Gal-3 GDF-15	IL-18, TNF-*α* MCP-1 GSN CYR61	GSN GDF-15	IL-6, 8, 18 TNF-*α* Gal-3 CYR61	

Other biomarkers	Hb FGF23	Hp2-2 Hepcidin-25 Renal oximetry Urinary PO_2_ MiR-21 UmtDNA	Hp2-2, Hb Hepcidin-25 Renal oximetry Urinary PO_2_ MiR-21 UmtDNA	UmtDNA FGF23	Hp2-2 Hb

CSA-AKI: cardiac surgery-associated acute kidney injury; AKI: acute kidney injury; CK-MB: creatine kinase-MB; H-FABP: heart fatty acid binding protein; NT-proBNP: N-tnd persistederminal prohormone of brain natriuretic; AngII: angiotensin II; NGAL: neutrophil gelatinase-associated lipocalin; TIMP-2: tissue inhibitor of metalloproteinases 2; IGFBP-7: insulin-like growth factor-binding protein 7; ApoM: apolipoprotein M; UMOD: urinary uromodulin; MMP-7: matrix metalloproteinase-7; IL: interleukin; TNF-*α*: tumor necrosis factor alpha; MCP-1: monocyte chemotactic protein-1; Gal-3: galectin-3; GSN: gelsolin; CYR61: cysteine-rich protein 61; GDF-15: growth differentiation factor 15; RFR: renal functional reserve; Alb: albumin; UA: uric acid; Hp2-2: 2-2 phenotype of haptoglobin; Hb: hemoglobin; PO_2_: oxygen tension; MiR-21: microRNA-21; UmtDNA: urinary mitochondrial DNA; FGF23: fibroblast growth factor 23.
